# Supplementary material for: The interactome of intact mitochondria by cross-linking mass spectrometry provides evidence for coexisting respiratory supercomplexes
Source: Mol Cell Proteomics. 2017 Dec 8;17(2):216–32. doi: 10.1074/mcp.RA117.000470 (PMC5795388; doi:10.1074/mcp.RA117.000470)
Supplement: Supplemental Data [file supp_RA117.000470_133922_0_supp_23978_fzffwf.zip › spectra_annotation/mito_spectra_annotation/350-2-5-1-19-1.pdf]

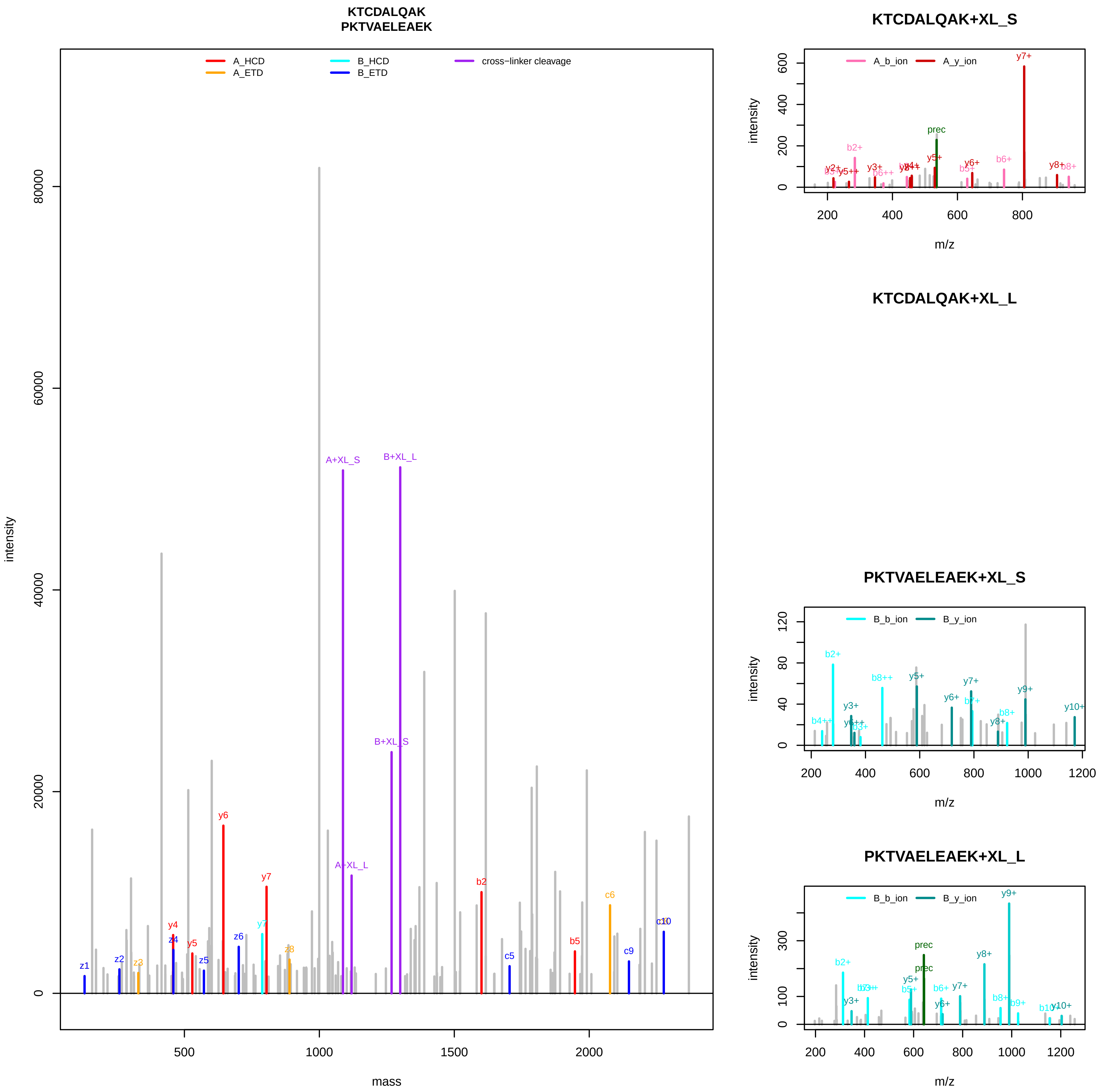
KTCDALQAK
PKTVAELEAEK
A_HCD
A_ETD
B_HCD
B_ETD
cross−linker cleavage
A+XL_S
B+XL_L
B+XL_S
A+XL_L
y4
y5
y6
y7
b2
b5
z1
z2
z3
z4
z5
z6
z8
c5
c6
c9
c10
intensity
mass
0
20000
40000
60000
80000
500
1000
1500
2000
KTCDALQAK+XL_S
A_b_ion
A_y_ion
prec
y7+
y8+
b8+
b6+
y6+
b5+
b2+
y3+
y5+
y2+
m/z
KTCDALQAK+XL_L
PKTVAELEAEK+XL_S
B_b_ion
B_y_ion
b2+
b8++
y5+
y7+
y6+
y9+
y10+
y3+
b8+
y8+
b7+
b4++
PKTVAELEAEK+XL_L
y9+
prec
y8+
b2+
y7+
y5+
b5+
b6+
y6+
y3+
b8+
b9+
b10+
y10+
m/z
intensity
